# XPO1-dependent nuclear export regulates NS3 localization and promotes DENV-2 replication through mitochondrial remodeling and interferon suppression

**DOI:** 10.1080/19491034.2026.2707719

**Published:** 2026-07-28

**Authors:** Selvin Noé Palacios-Rápalo, Jonathan Hernández-Castillo, Luis Adrián De Jesús-González, Daniel Talamas-Lara, Carlos Daniel Cordero-Rivera, Bulmaro Cisneros-Vega, Jose Manuel Reyes-Ruiz, Rosa María del Ángel

**Affiliations:** a Department of Infectomics and Molecular Pathogenesis, Center for Research and Advanced Studies (CINVESTAV), Mexico City, Mexico; b Laboratorio de Virología Molecular, Unidad de Investigación Biomédica de Zacatecas, Instituto Mexicano del Seguro Social, Zacatecas, México; c Unidad de Microscopía Electrónica, Laboratorios Nacionales de Servicios Experimentales (LaNSE), Centro de Investigación y de Estudios Avanzados del Instituto Politécnico Nacional (CINVESTAV), Mexico city, Mexico; d Department of Genetics and Molecular Biology, Center for Research and Advanced Studies (CINVESTAV), Mexico City, Mexico; e Unidad Médica de Alta Especialidad, Hospital de Especialidades No. 14, Centro Médico Nacional “Adolfo Ruiz Cortines”, Instituto Mexicano del Seguro Social (IMSS), Veracruz, Mexico; f Facultad de Medicina, Región Veracruz, Universidad Veracruzana, Veracruz, Mexico

**Keywords:** NS3 protein, dengue virus, nuclear export, Exportin 1, leptomycin B, mitochondrial elongation

## Abstract

Nucleocytoplasmic transport is essential for cellular homeostasis and is frequently exploited by viruses during infection. Although dengue virus (DENV) non-structural protein 3 (NS3) undergoes nuclear trafficking, the role of nuclear export pathways in its localization and in viral replication remains poorly understood. Here, we show that pharmacological inhibition of exportin 1 (XPO1) promotes the accumulation of DENV-2 NS3 in both the nucleus and mitochondria of infected Huh-7 cells. XPO1 inhibition also induces mitochondrial morphological alterations resembling those observed during DENV infection. Moreover, blockade of nuclear export enhances DENV-2 replication in interferon-stimulated cells by reducing type I interferon production, suggesting the establishment of a pro-viral cellular environment. Our findings reveal that XPO1-mediated nuclear export contributes to the regulation of NS3 localization and links nuclear export to mitochondrial remodeling and suppression of antiviral signaling during DENV infection.

## Introduction

Orthoflaviviruses, such as dengue virus (DENV) and Zika virus (ZIKV), are pathogens of major medical relevance and global distribution, causing a wide spectrum of clinical manifestations, ranging from self-limiting febrile illness to severe disease with hemorrhage and shock syndrome [1]. Their replication cycle occurs predominantly in the cytoplasm, associated with endoplasmic reticulum membranes, where viral translation and assembly of the replication complexes take place [2]. However, our group and others [3–5] have demonstrated that several viral components also interact with the cell nucleus, suggesting that this organelle plays a key role in modulating infection [6–9].

In particular, the non-structural proteins NS5 (RNA-dependent RNA polymerase, RdRp) and NS3 (protease – helicase) have been detected in the nucleus during the early stages of infection, where they may contribute to the modulation of nucleocytoplasmic trafficking and to alterations of the nuclear pore complex (NPC) [6]. Previous studies from our group have shown that DENV and ZIKV NS3 rapidly translocate to the nucleus of infected human and mosquito cells, subsequently redistributing to the cytoplasm [9,10]. We have also reported that ZIKV NS3 uses classical nuclear transport pathways, being imported via the importin-α/β route and exported through the CRM-1 exportin [11]. These findings highlight that orthoflaviviruses rely on tightly regulated nuclear transport mechanisms, which may represent potential therapeutic targets despite their cytoplasmic replication cycle.

The exchange of signaling molecules between the nuclear and cytoplasmic compartments is essential for cellular functions. This process is mediated by the NPC, which is composed of multiple copies of nucleoporins embedded across the nuclear envelope [12,13]. The integrity and function of the NPC can be compromised by viral proteases, including those from DENV and ZIKV, which cleave specific nucleoporins such as Nup153, Nup98, Nup62, and TPR, directly affecting the antiviral response and promoting viral replication [6].

In this context, we have also demonstrated that pharmacological inhibition of nuclear transport can exert antiviral effects. For instance, ivermectin blocks the importin-α/β pathway, reducing the nuclear localization of NS3 and NS5, while atorvastatin interferes with karyopherin-mediated transport [7]. The combined treatment significantly decreases DENV-2 infection in both in vitro and in murine models [7]. These findings underscore the potential of repurposing FDA-approved drugs to interfere with critical cellular processes hijacked by viruses during their life cycle.

However, most previous studies have focused on the nuclear import of NS3, and little is known about its export or about the potential connection between its nuclear retention and localization to other organelles. In this context, evidence exists that viral proteins can translocate to both the nucleus and mitochondria. For example, Hepatitis C virus (HCV) NS5A and core protein accumulate in the outer mitochondrial membrane due to the presence of a 10–amino acid mitochondrial targeting sequence (MTS), which is likely involved in apoptosis-related processes [14–16]. Moreover, DENV-1 NS3 possesses an MTS that allows its localization in the mitochondrial matrix, and GrpE protein homolog 1 (GrpEL1), a co-chaperone of mitochondrial Hsp70 (mtHsp70), has been identified as a cleavage target of this protein [17]. The close communication between the nucleus and the mitochondria [18] underscores the importance of exploring the relationship between nuclear export, mitochondrial dynamics, and viral infection in DENV and other medically relevant flaviviruses.

Although the NS3 nuclear and mitochondrial localization has been demonstrated [10,19], previous data showed that nuclear import inhibition reduces DENV NS3 nuclear localization and viral replication [7]; however, it is unclear whether nuclear export pathways are implicated in NS3 redistribution. In addition, it is known that DENV remodels the mitochondrial network and attenuates the antiviral response [20], processes that are influenced by nuclear export inhibition [21,22]; it is not yet known whether nuclear export inhibitors could affect mitochondrial morphology and the interferon response during DENV infection.

The classical nuclear export pathway is mediated by exportin 1 (XPO1/CRM1), which recognizes the leucine-rich nuclear export signal (NES) of cargo proteins or ribonucleoprotein complexes and transports them from the nucleus to the cytoplasm [23]. This protein regulates the subcellular distribution of both viral and cellular factors and influences processes such as mitochondrial biogenesis and the immune response [21,22,24]. The first evidence supporting the role of CRM1 in nucleocytoplasmic transport included its association with the nucleoporin CAN/Nup214 [25,26]; the observation that the drug leptomycin B (LMB) inhibits the nuclear export of the HIV regulatory protein Rev and its associated RNA, and the recognition that LMB exerts its cytotoxic effect by directly targeting CRM1 [27].

Leptomycin B is a potent inhibitor of nuclear export that irreversibly binds to cysteine 528 of XPO1 [28]. More recently, a novel class of Selective Inhibitors of Nuclear Export (SINE) compounds has been developed. These molecules bind to the NES-binding groove of XPO1 and form a reversible covalent bond with cysteine 528, making them less cytotoxic than LMB [29,30]. Selinexor (KPT-330) is the best-known first-generation SINE compound. It has been tested in clinical trials, both as a single agent and in combination with other therapies, in solid tumors and hematological malignancies [30,31], as well as for its potential antiviral activity against viruses such as SARS-CoV-2 [32].

In the present study, we investigated the effects of XPO1-specific inhibitors (LMB and selinexor) on the mitochondria-nuclear localization of DENV-2 NS3, mitochondrial morphology, and biogenesis in Huh-7 cells, as well as viral replication and the interferon-dependent response. Together, our findings expand our understanding of the role of nucleocytoplasmic transport in NS3 biology and propose a novel scenario in which nuclear export inhibition may promote, rather than restrict, viral replication.

## Materials and methods

### Cell culture

The human hepatocellular carcinoma cell line Huh-7 was maintained in Advanced Dulbecco’s Modified Eagle Medium (DMEM) supplemented with 2 mM glutamine, 7% fetal bovine serum (FBS), high glucose (4 g/L), Amphotericin B (1000X), penicillin (50 U/mL), and streptomycin (50 μg/mL). Cells were grown at 37°C in a humidified atmosphere containing 5% CO_2_.

### Virus, stock production, and viral infection

DENV-2 (New Guinea C strain) was kindly provided by the Instituto de Diagnóstico y Referencia Epidemiológicos Dr. Manuel Martínez Báez (InDRE), Mexico. ZIKV (MEX_CIENI551 strain) was generously donated by Dr. Jesús Torres (Escuela Nacional de Ciencias Biológicas, Instituto Politécnico Nacional). Both viruses were propagated in CD-1 suckling mouse brains. Neonatal CD-1 (ICR-CD1 strain 022) were maintained under specific-pathogen-free conditions at the Laboratory Animal Production and Experimentation Unit (UPEAL-CINVESTAV). Protocols 0382–24 (for CD-1 mice) were approved by the Animal Care and Use Committee (CICUAL) at CINVESTAV-IPN, Mexico.

Briefly, five-day-old neonatal mice were intracranially infected with DENV-2 or ZIKV at 1 × 10^6^ particles/mL and allowed to infect for four days. Neonatal CD-1 mice were used exclusively for virus propagation and not as an experimental disease model. No experimental groups or treatment comparisons were performed in animals. The experimental unit was a single cage with 10 CD-1 mouse litters (each including one dam and her neonatal pups). Only the neonatal mice were infected intracranially. Individual animal numbers per group were not relevant as brains were pooled. No inclusion or exclusion criteria were applied, as all animals inoculated were processed for virus propagation. All mice were euthanized by hypothermia using an ice chamber, in accordance with approved institutional animal care guidelines, followed by freezing four days post-infection (dpi). The brains were then aspirated with a syringe, pooled, mashed, and centrifuged to remove cellular debris. The supernatant was filtered, aliquoted, and stored at −80°C. Brain extracts from uninfected CD-1 mice were processed in parallel and used as mock controls.

Huh-7 cells at ~80% confluence were infected with DENV-2 or ZIKV at a multiplicity of infection (MOI) of 5 in Hank’s Balanced Salt Solution for 2 h at 37°C. After adsorption, the inoculum was removed and replaced with complete medium. Infection was allowed to proceed for 24 h.

### Nuclear export inhibitors

Leptomycin B (LMB) (CHEMCruz, sc-358688) was resuspended in ethanol (vehicle) at 50 µg/mL and stored at − 20°C. Working concentrations were prepared from a 3.7 µM stock. Selinexor (KPT-330) was kindly provided by Dr. Bulmaro Cisneros Vega (Department of Genetics and Molecular Biology, CINVESTAV). Working concentrations were prepared from a 100 µM stock solution in DMSO (vehicle).

### Transfection of Huh-7 cells

The plasmid encoding DENV-2 NS2B3 (pcDNA_DENV2-NS2B3_V5) was obtained from Alan Rothman (Addgene plasmid #115906; RRID: Addgene_115906). The plasmid was propagated in DH5α-competent E. coli (Invitrogen) and purified using the Zippy Plasmid Miniprep Kit (Zymo Research) following the manufacturer’s protocol. Huh-7 cells (70–80% confluence) were transfected by electroporation as described by Hashemi et al. [33], with minor modifications. Briefly, 1 × 10^7^ cells were washed with cold 1× PBS and resuspended in 100 µL Opti-MEM containing 5 µg of plasmid DNA. The cell – DNA suspension was transferred to a 4-mm Gene Pulser cuvette and electroporated using a Gene Pulser Xcell (Bio-Rad, Germany) at 170 V and 40 ms (exponential decay). Cells were then plated in Advanced DMEM supplemented with 15% FBS and incubated for 48 h, after which transfection efficiency was assessed by confocal microscopy.

### Subcellular fractionation

Huh-7 cells grown in 6-well plates at 80–90% confluence were mock-infected or infected with DENV-2 for 24 h. Cells were washed three times with 1× PBS and fractionated using the Abcam Cell Fractionation Kit (ab109719) according to the manufacturer’s instructions. Cytoplasmic and nuclear fractions were stored at − 80°C until analysis. Protein concentration in cytoplasmic extracts was determined using the BCA Protein Assay Kit (Thermo Scientific).

### Western blotting

A total of 30 µg of protein per sample was separated by SDS – PAGE, transferred onto nitrocellulose membranes (Bio-Rad), and blocked for 1 h at room temperature (RT) with 10% nonfat milk in PBST (1× PBS, 0.01% Triton X-100). Membranes were incubated with the following primary antibodies: mouse anti-XPO1 (Santa Cruz, sc-373865), anti-TOMM22 (Invitrogen, MA1-20161), anti-PGC1α (Invitrogen, PA5-72948), anti-Cyclin B1 (Santa Cruz, sc-245), anti-GAPDH (Santa Cruz, sc-47724; loading control), anti-lamin A/C (Santa Cruz, sc-376248; nuclear control), anti-calreticulin (Santa Cruz, sc-6468; cytoplasmic control), and rabbit anti-NS3 (GeneTex, GTX124252; infection control). HRP-conjugated goat anti-mouse or anti-rabbit IgG (1:5000; Cell Signaling) diluted in 5% nonfat milk/PBST was used as the secondary antibody. Proteins were visualized using the SuperSignal West Femto Chemiluminescent Substrate (Thermo Scientific), and densitometric analysis was performed with ImageJ.

### Confocal microscopy

Huh-7 cells grown to 70–80% confluence on coverslips in 24-well plates were mock-infected or infected with DENV-2 for 24 h. Cells were washed three times with 1× PBS, fixed with 4% paraformaldehyde (PFA) for 30 min, and permeabilized with 0.2% saponin, 1% FBS in 1× PBS for 30 min. Cells were incubated overnight at 4°C with rabbit polyclonal anti-NS3 antibody (1:300; GTX124252). Secondary antibodies included goat anti-mouse Alexa Fluor 488 and goat anti-rabbit Alexa Fluor 555 (Life Technologies). Nuclei were counterstained with Hoechst (Santa Cruz). Images were acquired using a Leica TCS SP8 confocal microscope (Leica Microsystems), and processed using LAS X Core Offline v3.3.0 software.

### Transmission electron microscopy (TEM)

Mock- or DENV-2–infected Huh7 cells (MOI = 5) were fixed with 2.5% glutaraldehyde in 0.1 M sodium cacodylate buffer (pH 7.2) for 1 h at room temperature (RT), followed by fixation with 1% osmium tetroxide for 1 h at RT. Samples were dehydrated through a graded ethanol and propylene oxide series, embedded in Polybed epoxy resin, and polymerized at 60°C for 24 h. Ultrathin sections (70 nm) were stained with uranyl acetate and lead citrate and examined using a JEOL JEM-1011 transmission electron microscope (JEOL Ltd., Tokyo, Japan).

### Transmission immunoelectron microscopy (TIM)

Mock-, DENV-2–, or ZIKV-infected Huh7 cells (MOI = 5; 12 or 24 hpi) were fixed with 4% paraformaldehyde/0.5% glutaraldehyde for 1 h at RT, dehydrated in an ethanol gradient, and embedded in LR White acrylic resin. Polymerization was performed under UV light at 4°C overnight. Resin sections (70 nm) mounted on Formvar-coated nickel grids were blocked in PBS with 10% FBS for 1 h, incubated with rabbit anti-NS3 antibody (1:20 in PBS with 5% FBS), washed, and incubated with 20-nm colloidal gold – conjugated anti-rabbit IgG (Ted Pella Inc.) for 1 h at RT. Sections were counterstained with uranyl acetate and lead citrate and examined using a JEOL JEM-1011 TEM.

### Mitochondrial morphometric analysis

Images obtained by confocal microscopy of cells treated with nuclear export inhibitors were segmented in Fiji/ImageJ using the pipelines described by Chaudhry et al. [34]. TOMM22-positive structures were preprocessed using the following commands: 1) ‘subtract background’ to remove background noise; 2) ‘sigma plus filter’ to reduce noise and smooth the object signal while preserving edges; 3) ‘enhance local contrast’ to enhance dark areas and minimize noise amplification; and 4) ‘gamma correction’ to correct any remaining dark areas. The resulting binary image was used as input for the ‘analyze particles’ command, which measured the ‘area’ and ‘perimeter.’ For network connectivity analysis, the ‘skeletonize 2D/3D’ command was applied to the image with a threshold to generate a skeleton map, and the ‘analyze skeleton’ command was used to calculate the number of branches, branch lengths, and branch junctions in the skeletonized network. For TEM images, the protocol reported by Lam et al. [35] was used in Fiji/ImageJ. Briefly, to record and track measurements, we opened the ‘Region of Interest (ROI) Manager’ tool. Next, to set the measurements that ImageJ will perform – such as area, circularity, and perimeter – we used the ‘Set Measurements’ command. Then, using the ‘Freehand’ tool, we traced the outline of the entire cell to save the measurement, and clicked ‘Add’ in the ‘ROI Manager.’ This was used to normalize subsequent measurements. After that, we traced the outer mitochondrial membrane of each mitochondrion and added the shape to the ‘ROI Manager.’ By clicking ‘Measure’ in the ‘ROI Manager,’ we obtained the mitochondrial area measurements.

### Relative mRNA quantification

Mock- and DENV-2–infected Huh7 cells were used to evaluate PGC-1α and interferon-related gene expression. Total RNA was extracted using TRIzol reagent (Invitrogen) and treated with DNase I (New England Biolabs). From 50 ng of total RNA, qPCR was performed to quantify PGC-1α, IFN-β, and IFI44L transcripts using the following primers:

- **PGC-1α**: F 5'-ATCCTCTTCAAGATCCTGCT-3'; R 5'-GACTCTCGCTTCTCATACTCTC-3'
- **IFN-β**: F 5'-GATGAACTTTGACATCCCTGAG-3'; R 5'-TAGCAAAGATGTTCTGGAGCA-3'
- **IFI44L**: F 5'-TGATTGCAGTGAGGTTCTTC-3'; R 5';-GAAGCATAATTTCCAACCATC-3'

Each reaction contained 5 µL of qPCR SyGreen 1-Step Go Hi-ROX (PCR Biosystems), 0.5 µL of each primer at 10 µM, and 1–2 µL of cDNA diluted in RNase-free water. Cycling conditions were: 50°C for 10 min; 95°C for 2 min; 40 cycles of 95°C for 5 s and 60°C for 30 s, using the Illumina Eco system. Threshold values were set using mock-infected samples and non-template controls. Data were analyzed with EcoStudy v5.04890 and relative expression calculated using the 2^–ΔΔCT method [36].

### Quantification of IFN-β in supernatants

Huh7 cells were pre-stimulated with 20 µg/mL recombinant IFN-β for 6 h, then infected with DENV-2 or left uninfected in the presence of LMB or selinexor for 24 h. Subsequently, 500 µL of supernatant from each condition was collected and stored at −20°C until analysis. IFN-β levels were quantified in 50 µL of supernatant using the human IFN-β quantitative ELISA (Biotechne, Cat# DIFNB0), following the manufacturer’s instructions. IFN-β concentrations (pg/mL) were calculated from a standard curve using a MultiSkan SkyHigh plate reader (Thermo Scientific).

### Flow cytometry assay

Treated and untreated infected cells were analyzed by flow cytometry to determine the percentage of infected cells using anti-NS3 (GTX, 1:500) and anti-TOMM22 (1:1000) antibodies, as well as to quantify fluorescence intensity after LMB or selinexor treatment. A goat anti-mouse Alexa Fluor 488 antibody (Life Technologies) was used as a secondary antibody. Samples were acquired on a BD LSRFortessa, and data were analyzed using FlowJo v10. Three independent experiments were performed in duplicate to determine infection percentages.

### Viral titration

Supernatants from treated and untreated infected cells were used to determine viral titers. DENV titers were measured by plaque-forming unit (PFU) assay, and ZIKV titers were determined by focus-forming unit (FFU) assay. Three independent experiments were performed in duplicate for each assay.

### Quantification and statistical analysis

Image analysis was performed using Icy (an open-source platform from the Pasteur Institute) and Fiji/ImageJ (NIH website). For each condition, at least 3 independent experiments were performed; in each experiment, 3–5 representative fields were acquired under identical acquisition settings. Nuclear and cytoplasmic regions of interest (ROIs) were manually delimited using Hoechst and cell boundaries, respectively. The mean fluorescence intensity (MFI) for each ROI was used to calculate the nucleus-cytoplasm fluorescence ratio (Fn/C) as follows: Fn/C=(Fn−Fb)(Fc−Fb), where Fb represents background fluorescence, Fn represents nuclear fluorescence, and Fc represents cytoplasmic fluorescence. MFI values for Fn/C were expressed as mean ± standard error of the mean (SEM). Statistical analyses were performed using Student’s *t*-test to compare mock- and virus-infected cells. For mRNA quantification, ordinary one-way ANOVA followed by Tukey’s multiple-comparisons test was used. Differences were considered statistically significant at *p* < 0.05.

### Ethics statement

This study was performed following the Official Mexican Standard Guidelines for Production, Care, and Use of Laboratory Animals (NOM-062-ZOO-1999). The protocol, number 0382–24, was approved by the Animal Care and Use Committee (CICUAL) at CINVESTAV-IPN, Mexico.

### Data availability statement

The data supporting the findings of this study can be found https://data.mendeley.com/datasets/zygzyvfj6k/1

## Results

### The NS3 protein of DENV-2 is localized in the cytoplasm, nuclei, and mitochondria of Huh-7 cells

Due to its multifunctional nature, the orthoflavivirus NS3 protein has been extensively studied in relation to its subcellular localization. NS3 from DENV-1 has been shown to localize in the cytoplasm, nucleus, and mitochondria, owing to the presence of both nuclear and mitochondrial targeting sequences [19]. Because NS3 localizes to both the nucleus and mitochondria, and given the role of nuclear – mitochondrial communication in regulating cellular stress and antiviral responses, we investigated whether inhibition of XPO1-dependent nuclear export alters NS3 distribution between these compartments.

**Figure 1.**
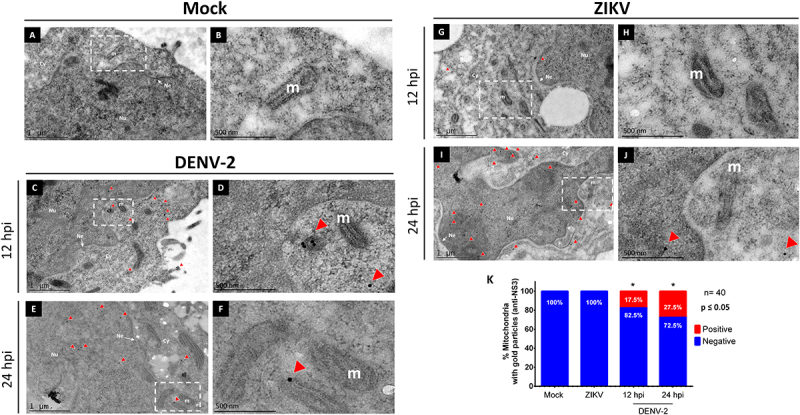
Subcellular localization of DENV-2 and ZIKV NS3 protein by immunogold TEM. (A – F) Transmission immunoelectron microscopy (TIM) images of Huh-7 cells infected with DENV-2 at 12- and 24-hours post-infection (hpi), showing NS3 protein detected by 20-nm immunogold particles (red arrows) localized in the cytoplasm, nucleus, and mitochondria. Mitochondria (M), nucleus (N), nuclear envelope (Ne, white arrow), and cytoplasm (C) are indicated. (G–J) Representative TIM images of Huh-7 cells infected with ZIKV at the same time points, showing NS3 immunogold labeling in the cytoplasm and nucleus, but not in mitochondria. (K) Quantification of the percentage of mitochondria positive for NS3 immunogold labeling in DENV-2– or ZIKV-infected cells at 12 and 24 hpi. Data represent mean ± SEM from three independent experiments. *p < 0.05.

Here, using transmission immunoelectron microscopy (TIM) of DENV-2–infected Huh-7 cells, we detected gold particles corresponding to NS3 within mitochondria at 12 and 24 hours post-infection (hpi) (Figure 1(A–F)). As expected, NS3 was also observed in the cytoplasm and nuclei of infected cells from 12 hpi onward, consistent with earlier reports [10]. To compare DENV and ZIKV, we infected Huh-7 cells with ZIKV and found gold particles (NS3) in the cytoplasm and nucleus but not in mitochondria (Figure 1(G–J)). Quantification revealed that 100% of mitochondria were NS3-negative in ZIKV-infected cells, whereas 17.5% and 27.5% of mitochondria were NS3-positive in DENV-2–infected cells at 12 and 24 hpi, respectively (Figure 1(K)).

Given this differential mitochondrial localization, we hypothesized that ZIKV NS3 may lack the mitochondrial targeting signal (MTS) reported for DENV-1 [17]. Multiple sequence alignment showed that this MTS is conserved among DENV serotypes but is not sufficiently conserved in ZIKV NS3 or in other flaviviruses analyzed (Table 1). These results suggest that mitochondrial localization of NS3 is a feature conserved among DENV serotypes but not in ZIKV, suggesting distinct targeting properties. In contrast, conserved nuclear import and export sequences are present in both viruses [7,11], supporting the observed nuclear localization of NS3 in DENV and ZIKV.

**Table 1.** Multiple alignment of the mitochondrial transport sequence (MTS) in NS3 protein sequences of *Orthoflaviviruses.*

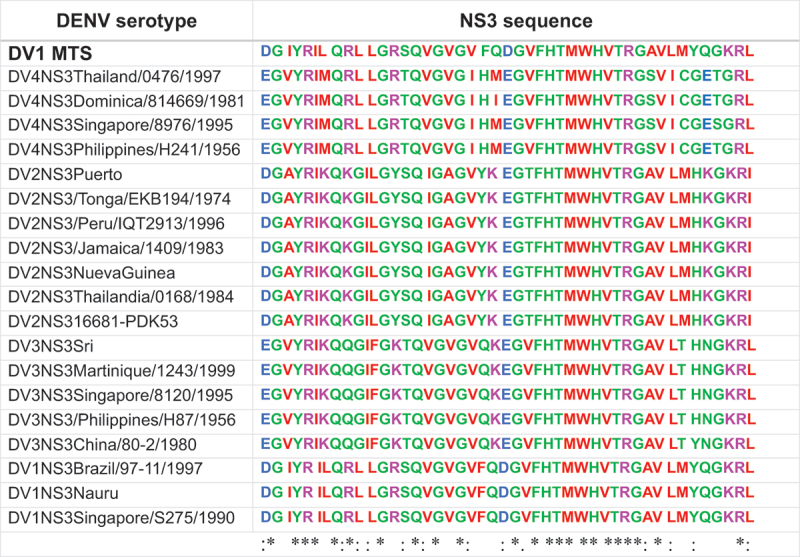
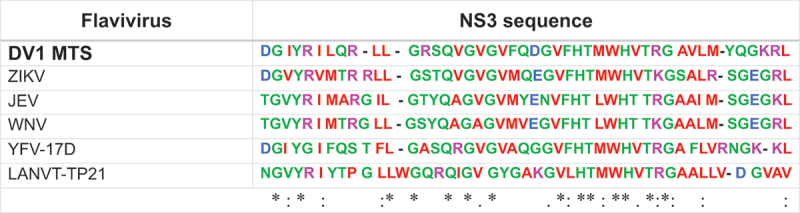

### NS3 protein increases in the nucleus and mitochondria upon inhibition of XPO1-dependent nuclear export

To determine whether XPO1-mediated nuclear export influences NS3 distribution between cellular compartments, we inhibit XPO1-dependent export by using 37 nM leptomycin B (LMB) for 24 h, based on viability assays (Fig. S1A). To validate this inhibition, we examined the subcellular localization of cyclin B1 (CNB1), which contains an XPO1-dependent nuclear export signal.

**Figure 2.**
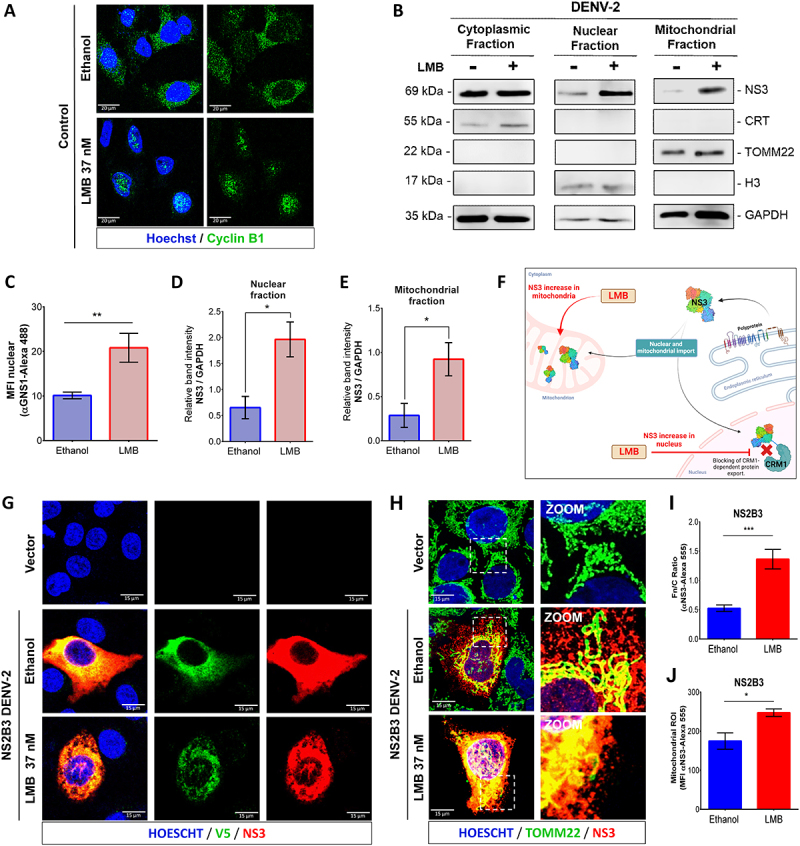
Nuclear and mitochondrial up-regulation of NS3 protein following exportin-1 inhibition. (A) Indirect immunofluorescence (IIF) analysis of cyclin B1 (CNB1) in Huh-7 cells treated with vehicle (ethanol) or leptomycin B (LMB, 37 nM) for 24 h, showing cytoplasmic localization under control conditions and nuclear retention upon XPO1 inhibition. (C) Mean fluorescence intensity (MFI) quantification of CNB1 in the nuclear region of LMB mock-infected cells indicates a significant increase relative to control. (B, D, and E) Western blot (WB) of cytoplasmic and nuclear fractions from DENV-2–infected Huh-7 cells treated with LMB or vehicle, probed with anti-NS3 antibody. GAPDH and calreticulin (CRT) were used as cytoplasmic markers and histone H3 as a nuclear marker. Densitometric analysis shows increased NS3 in the nuclear fraction after LMB treatment. (E) WB of mitochondrial fractions demonstrating enhanced NS3 signal following LMB treatment; TOMM22 was used as a mitochondrial marker. (F) Schematic representation summarizing NS3 redistribution in response to XPO1 inhibition. (G – I) Confocal microscopy of Huh-7 cells transfected with NS2B3-V5 in the presence or absence of LMB, revealing increased nuclear NS2B3 accumulation. Mean fluorescence intensity (MFI) quantification of nuclear regions of interest (ROIs) shows a significant increase relative to the control. (G) Confocal colocalization of NS2B3 (red) with the mitochondrial marker TOMM22 (green). Pearson’s correlation coefficient (*r* = 0.7) indicates robust colocalization, and MFI quantification of mitochondrial ROIs confirms increased NS3 accumulation during LMB treatment. Data represent mean ± SEM from three independent experiments. Statistical significance was determined by a t-test. *p < 0.05; **p < 0.01; ***p < 0.001.

Indirect immunofluorescence (IIF) confocal microscopy showed that CNB1 was predominantly cytoplasmic in vehicle-treated cells (Figure 2(A)). treatment with 37 nM LMB caused CNB1 accumulation in the nucleus, which was confirmed by Western blot (WB) of nuclear and cytoplasmic fractions (Fig. S1B). Fraction purity was validated using calreticulin (CRT), histone H3, and GAPDH. These results confirm that 37 nM LMB effectively blocks XPO1-mediated export. Under these conditions, confocal imaging of DENV-2–infected cells revealed increased nuclear NS3 signal during LMB treatment compared with vehicle controls, consistent with the significant rise in mean fluorescence intensity (MFI) (Fig. S1C). WB analysis confirmed that nuclear export inhibition markedly increases NS3 levels in the nuclear fraction (Figure 2(B–D)). LMB also significantly elevated NS3 levels in the mitochondrial fraction (Figure 2(E)), and the mitochondrial fraction’s purity was validated by TOMM22 labeling. Together, these results indicate that pharmacological inhibition of XPO1 increases NS3 abundance in both nuclei and mitochondria of Huh-7 cells (Figure 2(F)).

To further determine whether the changes in NS3 localization observed during infection are intrinsic to NS3 or dependent on the full viral replication context, we analyzed cells expressing NS2B-NS3 (NS2B3) in the absence or presence of LMB. Confocal microscopy showed a significant nuclear increase of NS2B3 upon LMB treatment (Figure 2(G,I)). TOMM22 staining and Pearson’s correlation analysis revealed a positive NS2B3–TOMM22 correlation (*r* = 0.70), supporting mitochondrial localization (Fig. S1D). Consistent with TEM observations, ZIKV NS3 displayed a predominantly cytoplasmic distribution and did not show clear colocalization with mitochondria (Pearson’s correlation coefficient, *r* = 0.24) (Fig. S1E). LMB treatment also significantly increased NS2B3 MFI in mitochondrial regions of interest (ROI) (Figure 2(H,J)). Notably, NS2B3 transfection – with or without LMB – altered mitochondrial morphology (Fig. S1D). Collectively, these results demonstrate that DENV-2 NS3 nuclear export depends on the XPO1 pathway and that inhibition of this pathway enhances NS3 accumulation in both the nucleus and mitochondria of Huh-7 cells.

### Blocking the nuclear export induces mitochondrial elongation in Huh-7 cells in a similar way to DENV infection

To understand how inhibition of nuclear export influences NS3 mitochondrial localization, we considered a previous report showing that pharmacological modulation of XPO1 induces mitochondrial elongation in Hutchinson-Gilford progeria syndrome (HGPS) cells [21]. Because mitochondrial elongation is also a hallmark of DENV infection [20,37], we evaluated whether inhibiting XPO1-dependent nuclear export alters mitochondrial morphology in Huh-7 cells. We tested leptomycin B (LMB), an irreversible inhibitor of XPO1, and Selinexor (KPT-330), a selective inhibitor of nuclear export (SINE) compound with reversible binding properties. Selinexor toxicity was assessed by MTT assays, and 200 nM was selected for nuclear export inhibition (Fig. S2A). Confocal images confirmed that both 37 nM LMB and 200 nM selinexor promoted nuclear accumulation of cyclin B1 (CNB1), demonstrating effective XPO1 inhibition (Fig. S2B, C).

**Figure 3.**
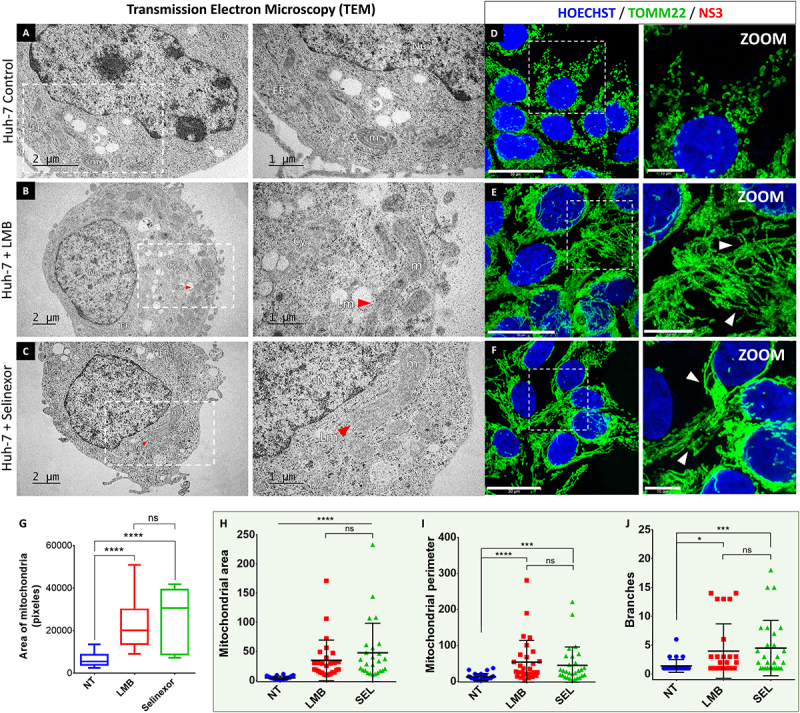
Mitochondrial morphometry in mock-infected Huh-7 cells treated with specific exportin-1 inhibitors. (A) Transmission electron microscopy (TEM) image of untreated (control) Huh-7 cells showing rounded or short mitochondria under basal conditions. (B–C) Representative TEM micrographs of Huh-7 cells treated for 24 h with leptomycin B (LMB, 37 nM) or selinexor (200 nM), showing elongated mitochondria (Lm, red arrows) relative to untreated controls. (D–F) Confocal microscopy of Huh-7 cells stained with anti-TOMM22 (green), revealing rounded mitochondrial morphology in control cells (D) and elongated mitochondrial (white arrows) networks after LMB (E) or selinexor (F) treatment. (G) Quantification of mitochondrial area from TEM images showing a significant increase in mitochondrial size in LMB- and selinexor-treated cells compared with controls. (H–J) Graphs of the two-dimensional (2D) morphometric analysis of mitochondria from confocal images (highlighted in the green rectangle) using ImageJ, demonstrating increased mitochondrial area (H), perimeter (I), and branching index (J) following XPO1 inhibition. Data represent mean ± SEM from three independent experiments. Statistical comparisons were performed within each condition using one-way ANOVA followed by Tukey’s multiple comparisons test. *p < 0.05; ***p < 0.001; ****p < 0.0001; ns, not significant.

Huh-7 cells treated with LMB or selinexor, or left untreated, were mock-infected or infected with DENV-2, and mitochondrial morphology was analyzed by transmission electron microscopy (TEM). Mock-infected control cells displayed mostly rounded or short mitochondria (Figure 3(A)). In contrast, cells treated with LMB or selinexor exhibited elongated mitochondria, although rounded mitochondria were still present (Figure 3(B,C)). Quantification showed a significant increase in mitochondrial area in both inhibitor treatments compared with controls (Figure 3(G)). Confocal microscopy confirmed these findings: TOMM22 staining showed rounded mitochondria in controls and elongated forms in both inhibitor-treated conditions (Figure 3(D–F)). Two-dimensional (2D) analyses further revealed increased mitochondrial area, perimeter, and branching in the presence of XPO1 inhibitors (Figure 3(H–J)), indicating that nuclear export blockade promotes mitochondrial elongation in Huh-7 cells.

**Figure 4.**
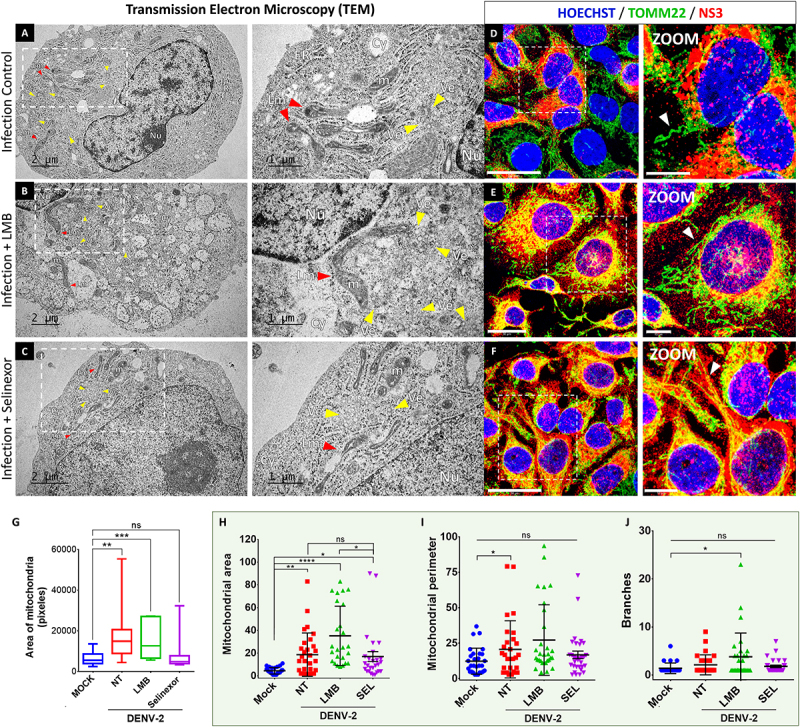
Mitochondrial morphometry in DENV-infected Huh-7 cells treated with exportin-1 inhibitors, extension of Figure 3. (A) Transmission electron microscopy (TEM) images of DENV-2-infected Huh-7 cells at 24 h post-infection (hpi), showing virus-induced vesicles (Ve, yellow arrows) and elongated mitochondria (red arrows) compared with mock-infected controls. (B–C) Representative TEM micrographs of infected cells treated with leptomycin B (LMB, 37 nM) or selinexor (200 nM), showing elongated and thinner mitochondria, as well as damaged mitochondria exhibiting disrupted cristae and matrix inclusions. (D–F) Confocal microscopy of infected Huh-7 cells stained with anti-TOMM22 (green) and anti-NS3 (red), showing mitochondrial elongation in untreated infected cells (D) and similar morphology following treatment with LMB (E) or selinexor (F). (G) Quantification of mitochondrial area from TEM images showing a significant increase in mitochondrial size in DENV-2-infected cells and in LMB-treated infected cells compared with mock controls. (H–J) Graphs of the two-dimensional (2D) morphometric analysis of confocal images (highlighted in the green rectangle) showing mitochondrial area (H), perimeter (I), and branching index (J) in DENV-2-infected cells treated with vehicle, LMB, or selinexor. Data are presented as mean ± SEM from three independent experiments. Statistical comparisons were performed within each condition using one-way ANOVA followed by Tukey’s multiple comparisons test. *p < 0.05; **p < 0.01; ***p < 0.001; ****p < 0.0001; ns, not significant.

During DENV-2 infection, TEM images revealed virus-induced vesicles (Figure 4(A), yellow arrows, extension of Figure 3) and elongated mitochondria (Figure 4(A), red arrows), consistent with previous reports. Similarly, elongated mitochondria were observed during LMB and selinexor treatment, although these appeared thinner than those in mock controls (Figure 4(BC)). Damaged mitochondria with matrix inclusions and cristae disorganization were detected in some infected and infected-treated cells (Figure 4(B,C)). Quantification showed increased mitochondrial area during infection compared with the mock control (data from the mock in Figure 3). Although both inhibitors tended to enhance mitochondrial size, only LMB induced a significant increase during DENV-2 infection (Figure 4(G)).

Confocal microscopy likewise showed elongated mitochondria in infected, LMB-treated, and selinexor-treated cells (Figure 4(D–F)), with all infected/treatment conditions displaying a larger mitochondrial area than mock controls (data from the mock in Figure 3), but no differences between infection and inhibitor treatments (Figure 4(H)). 2D analysis confirmed that both inhibitors, despite their distinct mechanisms of action, slightly increased mitochondrial perimeter and branching during infection (Figure 4(I,J)). Selinexor also recapitulated the LMB-induced enhancement of NS3 nuclear and mitochondrial localization (Figure 4(F)) and significantly reduced XPO1 expression in both mock- and DENV-infected cells (Fig. S2D, E), consistent with the reported ability of SINE compounds to promote XPO1 degradation [38]. Together, these results indicate that blocking nuclear export promotes mitochondrial elongation in Huh-7 cells, mimicking the morphological changes observed during DENV-2 infection. However, LMB and selinexor treatments do not further enhance or diminish the elongation phenotype induced by DENV-2 infection.

### Pharmacological modulation of the XPO1 pathway increases NS3 protein expression and is associated with modest changes in mitochondrial biogenesis

**Figure 5.**
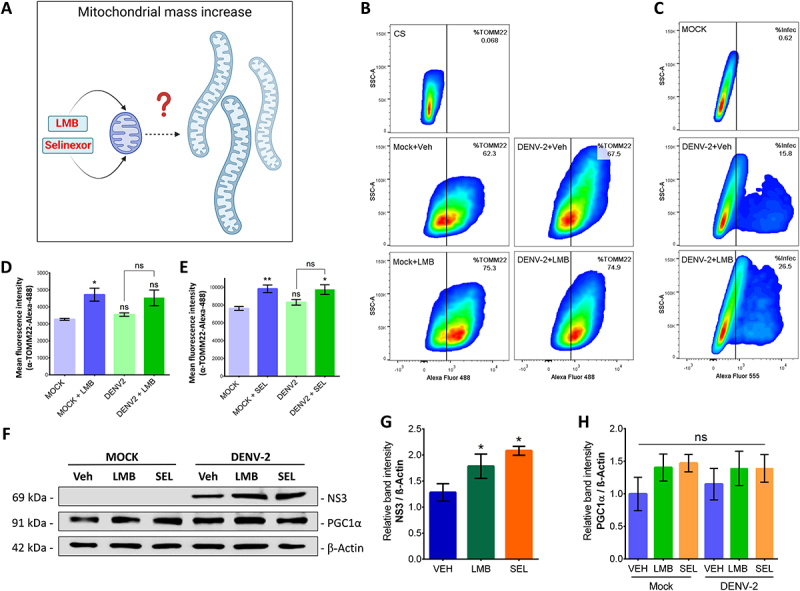
Inhibition of nuclear export by leptomycin B or selinexor increases mitochondrial content and NS3 protein levels. (A) Representative pseudocolor density plots showing TOMM22 fluorescence intensity in Huh-7 cells treated with vehicle, leptomycin B (LMB, 37 nM), or selinexor (200 nM), indicating changes in mitochondrial content. (B) Percentage of TOMM22-positive cells in mock-infected and DENV-2-infected populations. (C) Percentage of DENV-2-infected cells (anti-NS3–positive) under control and LMB treatment, showing increased infection frequency following nuclear export inhibition. (D–E) Mean fluorescence intensity (MFI) of TOMM22 in mock-infected (D) and DENV-2-infected (E) cells calculated from flow cytometry data using FlowJo software. (F–G) Western blot (WB) analysis of total cell extracts showing increased NS3 protein levels in cells treated with LMB or selinexor compared with untreated controls; GAPDH served as loading control. Densitometric quantification of NS3 levels is shown in (G). (H) WB analysis of PGC1-α expression in mock- and DENV-2-infected cells treated with LMB or selinexor, showing a trend toward increased levels consistent with mitochondrial biogenesis activation. Data represent mean ± SEM from three independent experiments. Statistical comparisons were performed within each condition using one-way ANOVA followed by Tukey’s multiple comparisons test. *p < 0.05; ns, not significant.

Given the mitochondrial elongation observed after nuclear export inhibition, we investigated whether LMB or selinexor also increased mitochondrial mass/content (Figure 5(A)). Previous studies reported that LMB promotes mitochondrial biogenesis in Hutchinson-Gilford progeria syndrome (HGPS) cells [21]. Therefore, we quantified mitochondrial content in LMB-treated Huh-7 cells by measuring TOMM22 mean fluorescence intensity (MFI) by flow cytometry, which reflects mitochondrial abundance at the population level but does not distinguish between changes in mitochondrial number, size, or morphology. Approximately 60–70% of cells were positive for TOMM22 in both treated and untreated conditions (Figure 5(B)). Infection analysis showed that at 24 hpi, 15% of vehicle-treated cells and 26% of LMB-treated cells were positive for NS3 (Figure 5(C)). A multiple comparison of the mean fluorescence intensity of TOMM22 across the groups revealed a significant increase in mock-infected cells treated with LMB or selinexor and a slight, non-significant increase in DENV-2-infected treated cells (Figure 5(D,E)), suggesting that inhibiting nuclear export may increase mitochondrial content in Huh-7 cells, consistent with the mitochondrial elongation and network redomdeling observed by TEM and confocal microscopy.

To explore this effect further, we examined the impact of nuclear export inhibition on the expression of PGC1-α, the master regulator of mitochondrial biogenesis, given that XPO1 modulation can induce PGC1-α upregulation [39]. Western blot analyses of mock- and DENV-2-infected cells treated with LMB or selinexor revealed a significant increase in NS3 protein levels compared with controls (Figure 5(F,G)), consistent with the increased percentage of infected cells (Figure 5(C)). We observed a non-significant trend toward increased PGC1-α protein expression in both mock-infected and infected treated cells (Figure 5(H)). In line with this, PGC1-α mRNA levels increased in mock-infected treated cells but not in infected cells (Fig. S3A). Collectively, these data suggest that inhibition of the XPO1 pathway with LMB or selinexor is compatible with a modest induction of mitochondrial biogenesis-related pathways; however, the contribution of PGC1-α remains preliminary under our experimental conditions.

### Inhibiting nuclear export is pro-viral for DENV in Huh-7 cells

Because inhibiting nuclear export accelerates DENV particle production [24], we expected LMB treatment to increase viral protein expression. Additionally, forced mitochondrial elongation has been shown to expand DENV replication factories [20]. Since nuclear export inhibitors also altered mitochondrial morphology in our system, we examined whether LMB or selinexor affected DENV-induced vesicle formation and viral replication.

**Figure 6.**
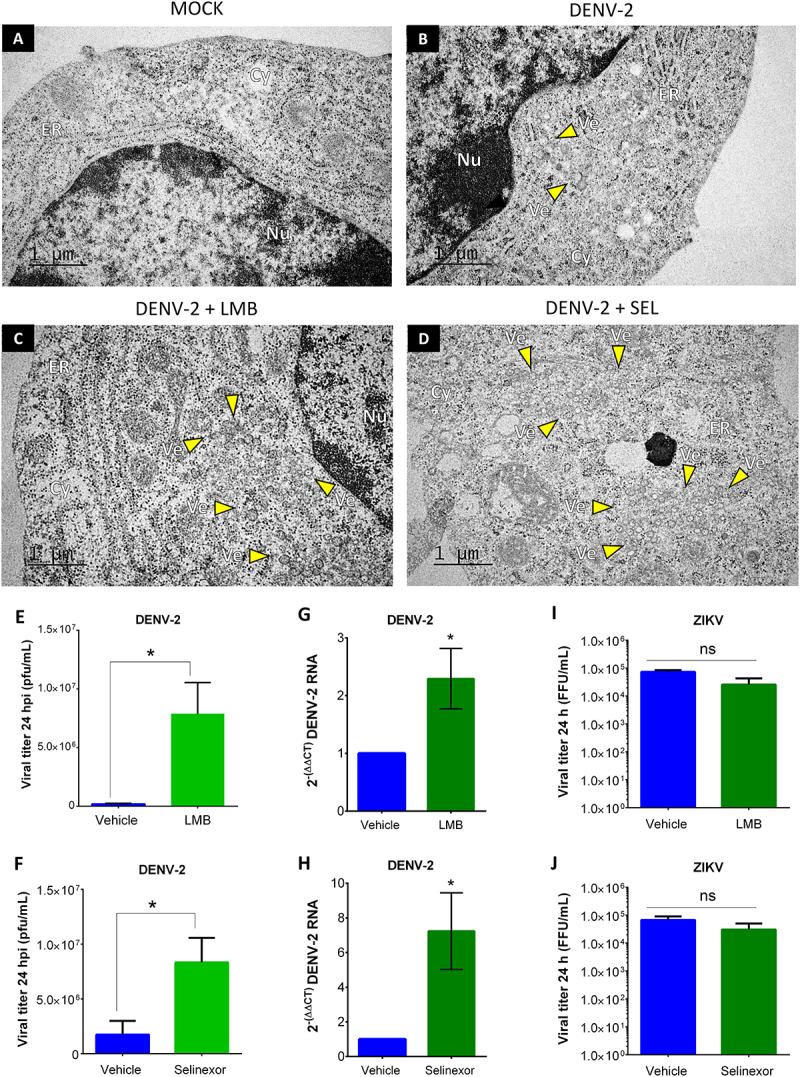
Nuclear export inhibition by SINE compounds enhances DENV infection. (A–D) Transmission electron microscopy (TEM) images of DENV-2-infected Huh-7 cells treated with vehicle (A), leptomycin B (LMB, 37 nM; B), or selinexor (200 nM; C–D), showing an increased number of virus-induced vesicles (Ve, yellow arrows) in the cytoplasm of treated cells compared with controls. (E–F) Quantification of infectious DENV-2 particles released into the supernatant, determined by plaque-forming unit (PFU) assay. Both LMB (E) and selinexor (F) significantly increased viral titers relative to vehicle-treated cells. (G–H) Quantification of intracellular DENV-2 RNA by RT-qPCR showing ~2-fold (LMB) and ~6-fold (selinexor) increases in viral genome copies compared with controls. (I–J) Viral replication analysis of ZIKV-infected Huh-7 cells treated with LMB or selinexor, showing no significant changes in viral titers, indicating that the enhancement is specific to DENV. Data represent mean ± SEM from three independent experiments performed in duplicate. Statistical significance was evaluated using a t-test. *p < 0.05; ns, not significant.

TEM analyses revealed an increase in virus-induced vesicles (Ve, yellow arrows) in infected cells treated with LMB or selinexor compared to vehicle-treated controls (Figure 6(A–D)). Consistently, LMB treatment significantly increased the viral titer in supernatants relative to controls (Figure 6(E)), and selinexor produced a similar increase (Figure 6(F)), despite their distinct effects on XPO1 protein levels (Fig. S2D-E). Quantification of viral RNA showed ~2-fold and ~6-fold increases in LMB- and selinexor-treated infected cells, respectively (Figure 6(G,H)). These results indicate that inhibition of the XPO1 pathway enhances the formation of virus-induced vesicles and promotes DENV-2 replication. In contrast, pharmacological inhibition of nuclear export during ZIKV infection did not significantly alter viral particle release or ZIKV RNA levels (Figure 6(I,J); Figure S3B – C).

### Inhibiting the XPO1 pathway increases DENV replication by preventing IFN production

**Figure 7.**
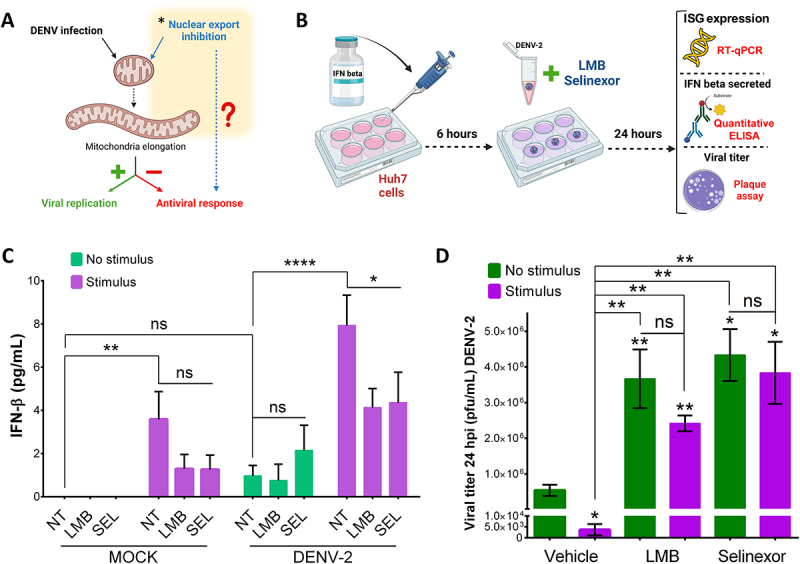
Nuclear export inhibitors dampen the antiviral response of IFN-stimulated Huh-7 cells, facilitating DENV replication. (A) Schematic representation illustrating the working hypothesis of this study. (B) Workflow diagram illustrating the quantification of IFN-β secretion, viral titers, and interferon-stimulated gene (ISG) expression in Huh-7 cells pre-stimulated with recombinant IFN-β (20 μg/mL, 6 h), infected with DENV-2 (MOI = 5), and treated with leptomycin B (LMB, 37 nM) or selinexor (200 nM) for 24 h. (C) Quantification of IFN-β protein levels in supernatants from pre-stimulated mock- and DENV-2-infected cells treated with LMB or selinexor, measured by ELISA. Cells infected, stimulated, and treated with nuclear export inhibitors (LMB or SEL) show approximately a 50% reduction in IFN-β secretion compared to cells infected, stimulated, and untreated (NT). No significant reduction in IFN-β secretion is shown in the stimulated and treated mock cells compared to the non-treated mock cells. Statistical comparisons were performed between NT, LMB, and Selinexor treatment within each infection and stimulation condition. (D) DENV-2 viral titers in supernatants of treated or untreated cells, showing a significant increase in infectious particles upon nuclear export inhibition, even under IFN-pre-activated conditions. Statistical comparisons were performed within each condition between treatments. Data represent mean ± SEM from three independent experiments performed in duplicate. Statistical comparisons were performed within each condition using one-way ANOVA followed by Tukey’s multiple comparisons test. Only relevant pairwise comparisons are shown. *p < 0.05; **p < 0.01; ***p < 0.001; ****p < 0.0001; ns, not significant.

DENV-induced mitochondrial elongation has been associated with enhanced viral replication and reduced activation of the interferon (IFN) response [20]. Given the mitochondrial alterations and increased DENV replication observed upon nuclear export inhibition, we hypothesized that LMB or selinexor may suppress the antiviral response, thereby facilitating DENV replication (Figure 7(A)). To evaluate this possibility, we first attempted to stimulate the IFN pathway using Poly I: C; however, Huh-7 cells did not show detectable activation (data not shown). Therefore, we pre-stimulated cells with recombinant IFN-β (20 μg/mL) for 6 h, as IFN-β can trigger a positive feedback loop that amplifies its own expression [40]. After stimulation, cells were infected with DENV-2 (MOI = 5) and treated with LMB or selinexor for 24 h. We then quantified the mRNA levels of IFN-β and the interferon-stimulated gene IFI44L, measured IFN-β protein in supernatants, and determined the viral titers (Figure 7(B)).

RT-qPCR analysis showed a ~ 2- to 4-fold increase in IFN-β and IFI44L mRNA expression in mock- and DENV-2-infected cells, regardless of stimulation status (Fig. S3D – E). IFN-β protein was only detectable by ELISA in pre-stimulated mock-infected and infected cells, and not in non-stimulated cells, even after treatment with LMB or Selinexor (Figure 7(C)). This discrepancy between mRNA expression and protein detection suggests that, although IFN-β transcription is induced, downstream processes such as mRNA export, translation, or secretion may be impaired under conditions of XPO1 inhibition. Notably, pre-stimulated infected cells treated with LMB or selinexor exhibited ~50% lower IFN-β levels than non-treated (NT) pre-stimulated controls (Figure 7(C)), indicating that nuclear export inhibition limits the efficiency of IFN production. In line with this, LMB or selinexor treatment significantly increased DENV-2 viral titers even when the IFN pathway was pre-activated. In contrast, IFN-β pre-stimulation reduced viral titers in non-treated infected cells (Figure 7(D)), confirming that DENV replication is sensitive to interferon-mediated antiviral signaling. Altogether, these results suggest that inhibiting the XPO1 pathway enhances DENV replication by suppressing IFN production, thereby counteracting the antiviral response.

## Discussion

Viruses hijack cellular machinery to support infection by exploiting organelle functions or modifying their composition [41]. Efficient use of organelles requires that viral components localize to specific subcellular compartments at defined stages of infection [42]. Although DENV proteins are translated in the endoplasmic reticulum and replication occurs in the cytoplasm, several viral proteins translocate to the mitochondria and the nucleus [19,43], suggesting that nucleocytoplasmic transport pathways play important roles in viral pathogenesis.

NS3 is a multifunctional protein involved in viral polyprotein processing and viral RNA replication [44]. Because it contains multiple targeting sequences, NS3 localizes to several cellular compartments, including the nucleus and mitochondria [10,11,17,19]. Accordingly, NS3 can degrade mitochondrial HSP70, nucleoporins of the nuclear pore complex (NPC), and the nuclear transcription factor EDRF1, contributing to pathological outcomes such as thrombocytopenia [6,7,19]. We previously showed that NS3 nuclear import occurs through the importin-α/β pathway and that its inhibition reduces NPC damage [7]. NS5 similarly relies on importin-α/β for nuclear import [45] and contains XPO1-regulated nuclear export sequences [24,46]. Whether XPO1 also mediates NS3 export had not been explored.

Here, we show that inhibition of XPO1 with LMB promotes nuclear accumulation of NS3 in Huh-7 cells during DENV-2 infection. The results obtained with NS2B3 expression indicate that NS3 localization is, at least in part, an intrinsic property of the protein that can be modulated by nuclear export. However, infection-specific factors are also likely to influence its distribution. Although we did not experimentally validate the predicted nuclear export signal (NES) of NS3, our findings are consistent with an XPO1-dependent export mechanism. ZIKV NS3 possesses a functional XPO1-regulated NES [11], and the same motif is present in DENV NS3 [10], supporting this interpretation.

We also detected NS3 in mitochondria by immunogold labeling. Although a mitochondrial targeting sequence (MTS) has been described for DENV NS3, this motif is not sufficiently conserved in other flaviviruses such as ZIKV. This suggests that mitochondrial localization of NS3 may be conserved among DENV serotypes but not across all flaviviruses. However, alternative explanations, including differences in replication kinetics, protein abundance, or infection efficiency between DENV-2 and ZIKV, cannot be excluded. Consistent with this, NS2B3 colocalized with TOMM22, and mitochondrial localization of DENV-2 NS3 increased upon nuclear export inhibition.

To further explore the relationship between nuclear export and mitochondrial dynamics, we examined mitochondrial morphology. Nuclear – mitochondrial communication is well established [18], and XPO1 modulation induces mitochondrial elongation in HGPS cells [21]. Consistent with these observations, both leptomycin B and selinexor induced mitochondrial elongation in Huh-7 cells. DENV infection itself induces mitochondrial elongation [20,37], as confirmed by both TEM and confocal microscopy. Although nuclear export inhibition did not further enhance mitochondrial elongation in infected cells, mitochondrial morphology remained significantly altered compared to controls. One possible explanation is that viral factors, such as NS4B-mediated modulation of mitochondrial dynamics [20,37], may limit additional effects of XPO1 inhibition during infection.

Mitochondrial remodeling is closely linked to organelle turnover, mitophagy, and biogenesis. In our study, XPO1 inhibition resulted in modest changes in mitochondrial content, compatible with a limited induction of mitochondrial biogenesis-related pathways [39]. Although PGC1-α is a key regulator of mitochondrial biogenesis, its contribution in our system remains preliminary. Consistent with previous reports, DENV infection did not significantly increase PGC1-α expression upon treatment. Therefore, additional studies will be required to determine whether nuclear export inhibition directly modulates mitochondrial biogenesis during infection [37,47].

Consistent with previous reports, DENV-2 replication increased following nuclear export inhibition [24]. Similarly, selinexor (KPT-330), a first-generation selective nuclear export inhibitor (SINE), enhanced viral replication, as evidenced by increased viral titers, RNA levels, NS3 expression, and virus-induced vesicles in Huh-7 cells. On the other hand, selinexor reduces SARS-CoV-2 spread by downregulating ACE2 [32], but enhances replication of murine hepatitis virus and SARS-CoV-2 in certain cell lines [48]. These findings underscore that XPO1 inhibition affects viral replication in virus- and cell-specific manners. Although both leptomycin B (LMB) and selinexor act on XPO1, their mechanisms of action differ. LMB irreversibly inhibits XPO1 through covalent modification, thereby blocking nuclear export without significantly affecting XPO1 protein levels [28]. In contrast, selinexor, a member of the SINE family, reversibly binds to XPO1 and has been shown to promote its proteasome-dependent degradation, leading to a reduction in protein expression [38,49]. Consistent with this, we observed a decrease in XPO1 levels in cells treated with selinexor, but not in those treated with LMB. Despite these differences, both inhibitors induced comparable effects on NS3 localization and mitochondrial morphology, suggesting that inhibition of XPO1 function, rather than its abundance, is the primary factor underlying the observed NS3 phenotype.

Importantly, selinexor and LMB suppress inflammatory cytokine production [32,50,51]. During DENV infection, LMB inhibits IL-8 secretion [24]. Our results show that both LMB and selinexor reduce IFN-β protein production despite increased mRNA levels, suggesting that nuclear export inhibition affects the interferon response at a post-transcriptional level. XPO1 is required for the export of mRNAs and regulatory proteins involved in antiviral responses [32]; therefore, its inhibition may impair translation or secretion of IFN-related factors. In addition, mitochondrial elongation induced by nuclear export inhibition may further disrupt antiviral signaling, contributing to a pro-viral environment [52]. Although we did not examine the localization of antiviral transcription factors, SINE compounds broadly retain these factors in the nucleus, impairing antiviral gene expression. In addition, DENV-induced mitochondrial elongation disrupts ER – mitochondria contact sites, which are required for innate signaling [20], thereby reducing type I/III IFN expression. Thus, mitochondrial elongation induced by nuclear export inhibition may exacerbate this antiviral blockade, further enhancing DENV replication and supporting redistribution of viral proteins among organelles. This suggests that the pro-viral effect arises from a combination of alterations in nucleocytoplasmic trafficking, mitochondrial remodeling, and attenuation of the IFN response, rather than from a single mechanism.

Together, these findings indicate that nuclear export plays a central role in regulating NS3 localization, mitochondrial dynamics, and antiviral responses during DENV infection, highlighting XPO1 as a key host factor influencing viral replication.

## Supplementary Material

Supplementary Information last.docx

## Data Availability

The data that support the findings of this study are available from the corresponding author (R.M.D.A.) upon reasonable request.
