# Supplementary material for: XPO1-dependent nuclear export regulates NS3 localization and promotes DENV-2 replication through mitochondrial remodeling and interferon suppression
Source: Nucleus. 2026 Jul 28;17(1):2707719. doi: 10.1080/19491034.2026.2707719 (PMC13418484; doi:10.1080/19491034.2026.2707719)
Supplement: Supplementary Information last.docx [file KNCL_A_2707719_SM3142.docx]

**Supplementary Information**

**XPO1 inhibition reshapes NS3 localization and is associated with mitochondrial remodeling, reduced interferon production, and increased DENV-2 replication**

Selvin Noé Palacios-Rápalo^1,7^, Jonathan Hernández-Castillo^1^, Luis Adrián De Jesús-González^2^, Daniel Talamas-Lara^3^, Carlos Daniel Cordero-Rivera^1^, Bulmaro Cisneros-Vega^4^, Jose Manuel Reyes-Ruiz^5,6^, and Rosa María del Ángel^1,^*

^1^ Department of Infectomics and Molecular Pathogenesis, Center for Research and Advanced Studies (CINVESTAV-IPN), Mexico City 07360, Mexico.

^2^ Laboratorio de Virología Molecular, Unidad de Investigación Biomédica de Zacatecas, Instituto Mexicano del Seguro Social, Zacatecas, Zacatecas, México.

^3^ Unidad de Microscopía Electrónica, Laboratorios Nacionales de Servicios Experimentales (LaNSE), Centro de Investigación y de Estudios Avanzados del Instituto Politécnico Nacional (CINVESTAV-IPN), Av. IPN 2508, Alcaldía Gustavo A. Madero (GAM), Mexico city 07360, Mexico.

^4^ Department of Genetics and Molecular Biology, Center for Research and Advanced Studies (CINVESTAV-IPN), Mexico City 07360, Mexico.

^5^ Unidad Médica de Alta Especialidad, Hospital de Especialidades No. 14, Centro Médico Nacional “Adolfo Ruiz Cortines”, Instituto Mexicano del Seguro Social (IMSS), Veracruz 91897, Mexico

^6^ Facultad de Medicina, Región Veracruz, Universidad Veracruzana, Veracruz 91700, Mexico.

^7^ Present address: Institut Pasteur, Structural Virology Unit, CNRS UMR3569, 75015, Paris, France.

***Correspondent author:**

Rosa María del Ángel, e-mail address: [rmangel@cinvestav.mx](mailto:rmangel@cinvestav.mx)


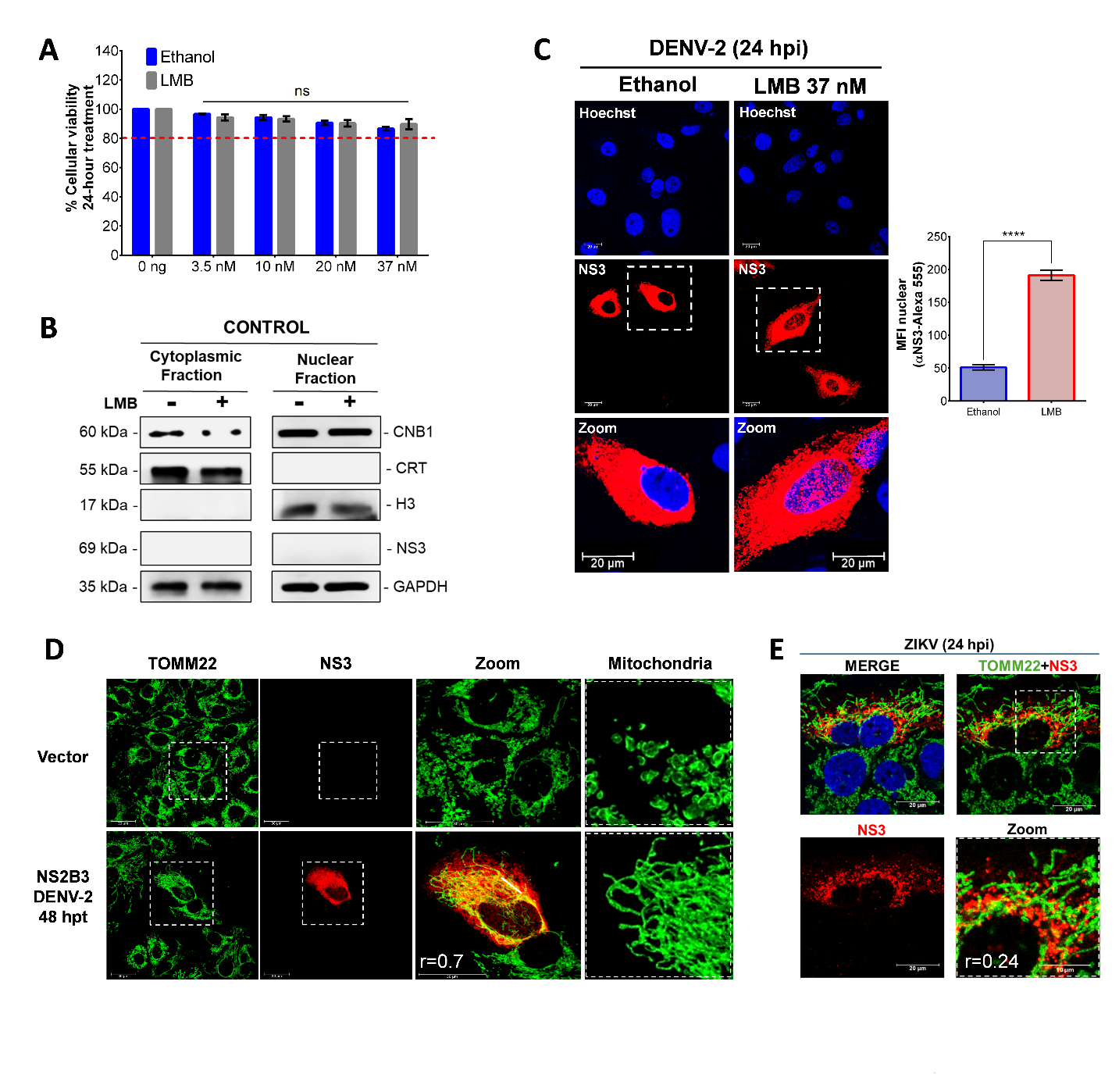
**Fig. S1. Percentage of cell viability and control of nuclear export inhibition following treatment with Leptomycin B.** (A) . Percentage of cell viability of LMB treatment at 24 h from two independent experiments in triplicated. Data were represented as mean ± standard error of the mean (SEM). Two-way ANOVA performed a statistical comparison with Sidak´s multiple comparison test. (B) Western blot of cytoplasmic and nuclear fractions from Mock–infected Huh-7 cells treated with LMB or vehicle, probed with anti-NS3 antibody. GAPDH and calreticulin (CRT) were used as cytoplasmic markers and histone H3 as a nuclear marker. (C) Confocal microscopy of Huh-7 cells infected with DENV-2 (24 hpi) in the presence or absence of LMB. Mean fluorescence intensity (MFI) quantification of nuclear regions of interest (ROI) indicates a significant increase relative to control. Data represent mean ± SEM from three independent experiments. Statistical significance was determined by t-test. ****p <0.0001. (D) Confocal microscopy of Huh-7 cells transfected with NS2B3 (48 hpt) and Pearson’s correlation analysis between TOMM22 (green) and NS3 (red) staining. (E) Confocal analysis of ZIKV NS3 (24 hpi) localization in Huh-7 cells and Pearson’s correlation analysis between TOMM22 (green) and NS3 (red) staining.

**
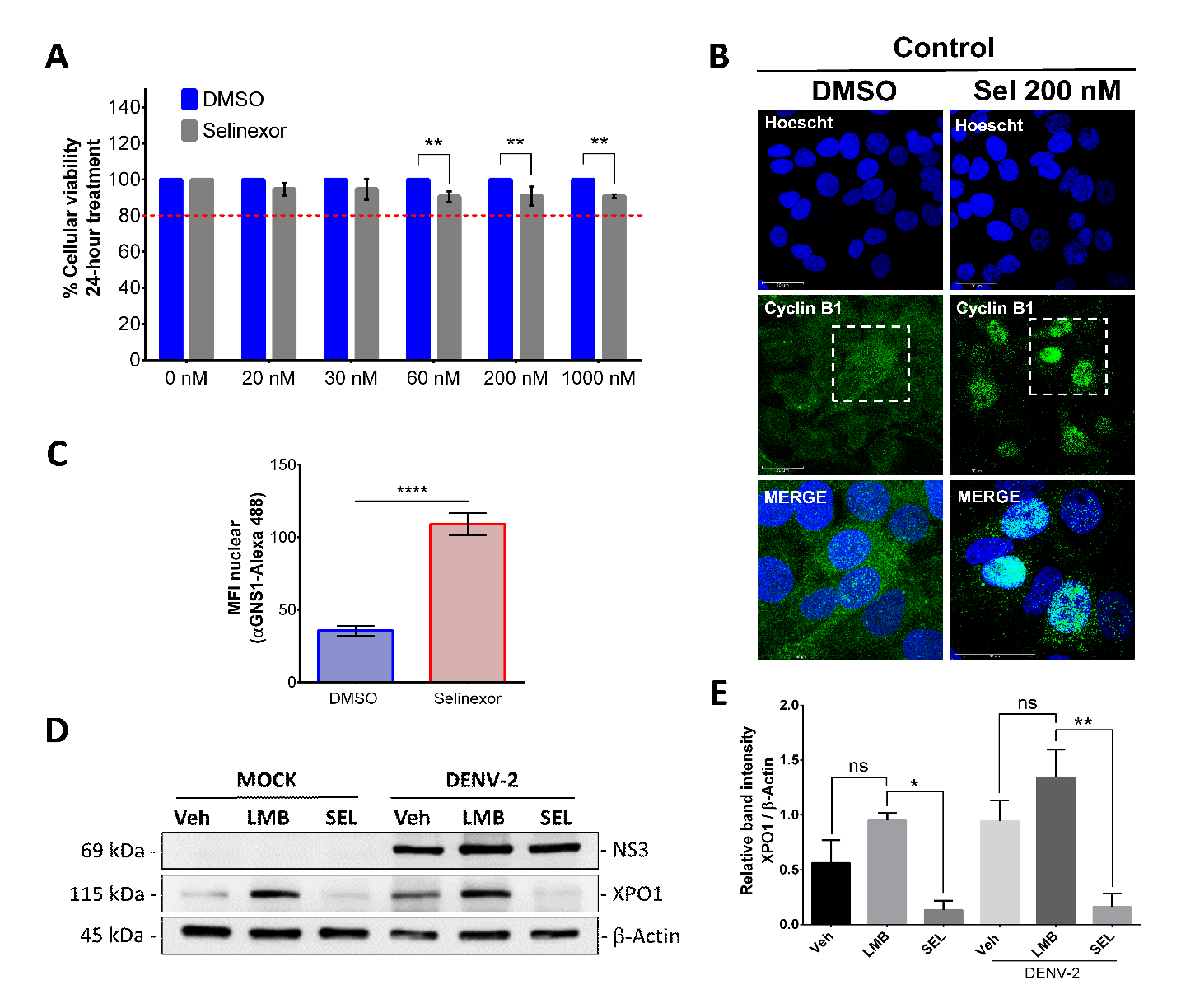
**

**Fig. S2. Percentage of cell viability and control of nuclear export inhibition following treatment with Selinexor.** (A) . Percentage of cell viability of Selinexor treatment at 24 h from two independent experiments in triplicated. Data were represented as mean ± standard error of the mean (SEM). Two-way ANOVA performed a statistical comparison with Sidak´s multiple comparison test. ∗∗p 0.0040. (B) Indirect immunofluorescence analysis of cyclin B1 (CNB1) in Huh-7 cells treated with vehicle (ethanol) or selinexor (200 nM) for 24 h, showing cytoplasmic localization under control conditions and nuclear retention upon XPO1 inhibition. (C) Mean fluorescence intensity (MFI) quantification of nuclear regions of interest (ROI) indicating a significant increase relative to control. Data represent mean ± SEM from three independent experiments. Statistical significance was determined by t-test. ****p <0.0001. (D and E) Western blot and densitometry of XPO1 derived from total extracts of mock-infected and DENV-2-infected cells treated with vehicle, LMB, and selinexor. Data represent mean ± SEM from three independent experiments. Statistical comparisons were performed within each condition using one-way ANOVA followed by Tukey’s multiple comparisons test. *p < 0.05; **p <0.01; ****p <0.0001; ns, not significant.

**
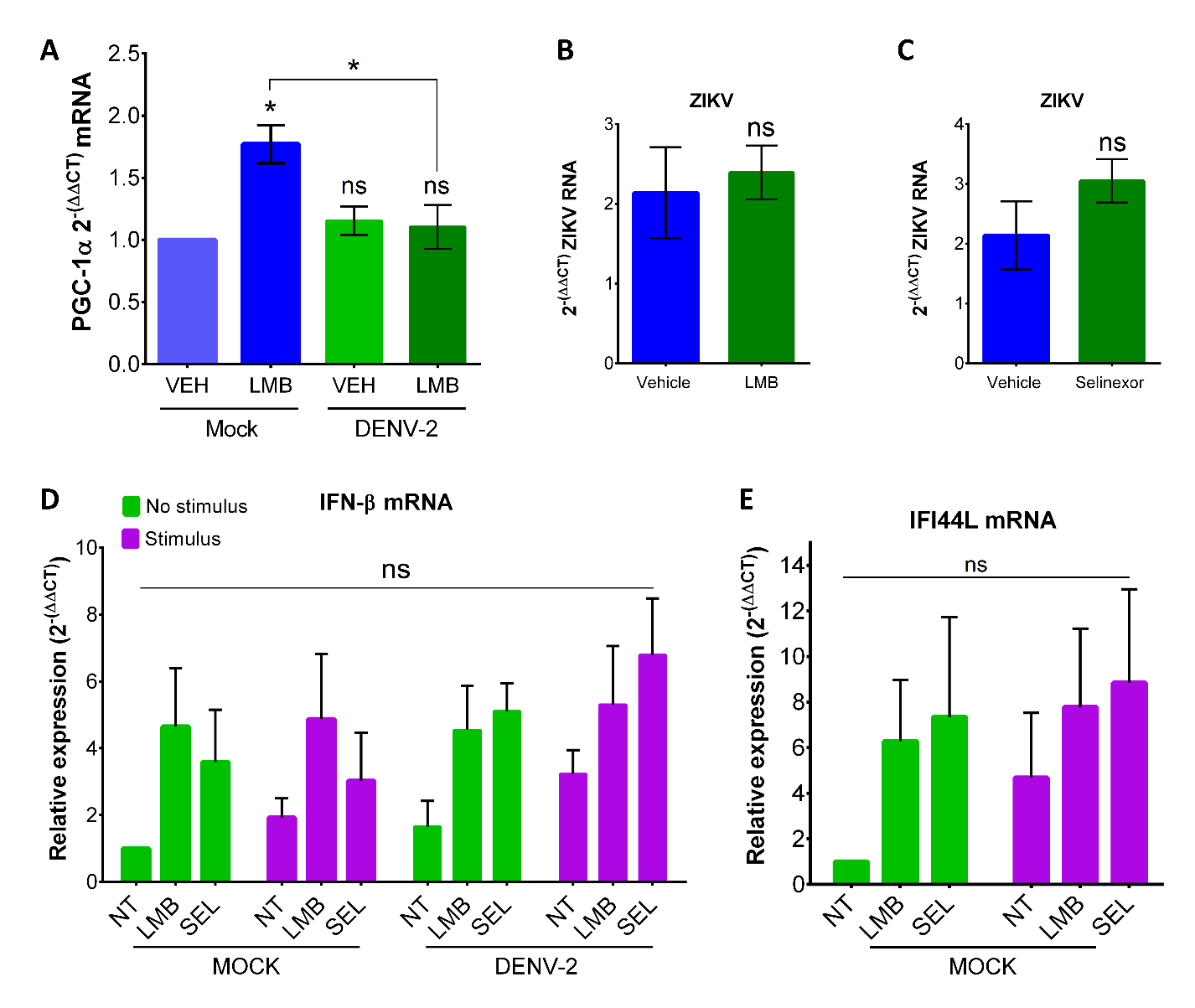
**

**Fig. S3. Relative expression of PGC1α, IFNβ, and IFI44L mRNA following treatment with LMB or selinexor.** (A) Expression levels of PGC1α transcript was determined by quantitative RT-PCR assays of mock and DENV-2-infected Huh7 cells treated with vehicle or LMB. (B and C) Viral replication analysis of ZIKV-infected Huh-7 cells treated with LMB or selinexor, showing no significant changes in viral RNA level. Data represent mean ± SEM from three independent experiments. Statistical significance was determined by t-test. ns, not significant.. (D and E) Expression levels of IFNβ and IFI44L transcripts were determined by quantitative RT-PCR assays of mock and DENV-2-infected Huh7 cells treated with vehicle, LMB or selinexor. Data represent mean ± SEM from three independent experiments performed in duplicate. Statistical comparisons were performed within each condition using one-way ANOVA followed by Tukey’s multiple comparisons test. *p < 0.05; ns, not significant.
